# The Challenge of Potential Drug–Drug Interactions Among People Living With HIV on Antiretroviral Therapy: A Cross-Sectional Study in Selected Provinces in China

**DOI:** 10.3389/fphar.2020.00800

**Published:** 2020-05-27

**Authors:** Huan Xia, Liying Gao, Xiaowen Gong, Silvere D. Zaongo, Tong Zhang, Hao Wu, Ping Ma, Xiaojie Huang

**Affiliations:** ^1^Department of Infectious Diseases, Nankai University Second People's Hospital, Tianjin, China; ^2^Department of Biostatistics, Tianjin Medical University, Tianjin, China; ^3^International School of Medicine, Tianjin Medical University, Tianjin, China; ^4^Department of Infectious Diseases, Beijing Youan Hospital, Capital Medical University, Beijing, China

**Keywords:** drug–drug interactions, human immunodeficiency virus, antiretroviral drugs, DDI, HIV, China

## Abstract

**Objectives:**

Potential drug–drug interactions (DDIs) are a significant therapeutic threat among human immunodeficiency virus (HIV)-positive individuals on antiretroviral (ARV) medications. DDIs involving ARV drugs in mainland China are unknown and insufficiently described. Herein, we investigated the prevalence and frequencies of potential ARV DDIs in Chinese people living with HIV (PLWH), then we assessed the risk factors associated with potential DDIs.

**Methods:**

This study was conducted with HIV-positive adults undergoing ARV medications from multiple centers across China. The latest prescription of each participant was evaluated for potential DDIs using the Liverpool HIV drug interaction database. Multivariable logistic regressions were used to evaluate the factors associated with DDIs.

**Results:**

Among 600 PLWH recruited, at least one non-HIV co-medication was observed in 511 (85.2%) individuals. A total of 2566 DDIs were identified, of which 11 (0.43%) and 311 (12.89%) were of contraindicated (red-flags) and dosage/timing adjustment required (orange-flags), respectively. Multivariate regression analysis revealed a higher risk of clinically significant DDIs (red- and orange-flagged comedication) associated with: the use of boosted protease inhibitors (*p* < 0.0001), boosted integrase strand transfer inhibitors (*p* < 0.0001), and non-nucleoside reverse transcriptase inhibitors-based ARV regimen (*p* < 0.0001); or the use of antiinfectives for systemic use (*p* < 0.0001), cardiovascular system drugs (*p* < 0.0001), nervous system drugs (*p* < 0.0001), fungal infection (*p* = 0.0071), and *Herpes simplex* virus infection (*p* = 0.0231).

**Conclusions:**

Potential DDIs and inappropriate medications constitute a burden for people living with HIV in China. The knowledge of DDIs patterns and the scan for DDIs is crucial. Indeed, they can help to prevent drug-related adverse outcomes in such immunodeficient population.

## Introduction

Globally, approximately 37.9 million people are living with human immunodeficiency virus (HIV)(Cambou and Landovitz, 2020). Although advances in treatment have yielded effective regimens, antiretroviral (ARV) agents are among the most therapeutically risky for drug-drug interactions (DDIs) presenting significant risks to people living with HIV (PLWH) and a challenge for clinicians to ensure appropriate and safe prescribing (Gong et al., 2019; Cambou and Landovitz, 2020). Concomitant medication use is more prevalent in PLWH than in the general population (Gimeno-Gracia et al., 2015). This setting is further accentuated by aging (Smith and Flexner, 2017; Ranzani et al., 2018; Back and Marzolini, 2020) and additional comorbidities such as opportunistic infections, non-acquired immune deficiency syndrome (AIDS) related comorbidities (Althoff et al., 2019), including non-AIDS malignancies, cardiovascular events, renal and hepatic diseases, and neurocognitive disorders often resulting in polypharmacy (usually defined as the simultaneous use of ≥ 5 drugs) (Courlet et al., 2019; Freedman et al., 2019; Edelman et al., 2020). Thus, the potential onset of DDIs becomes an essential concern for HIV-infected individuals with comorbidities that require concomitant medications, which can significantly alter the drugs exposure and their efficacy and toxicity (Seden et al., 2017). The most well-recognized DDIs mechanism is that many ARVs, especially pharmacologic boosters ritonavir or cobicistat-boosted protease inhibitors (PIs)-based and non-nucleoside reverse transcriptase inhibitors (NNRTIs), frequently lead to significant drug interactions since they may affect the cytochrome P450 enzymes or drug transporters as inhibitors, inducers or substrate involved with the metabolism of many commonly used medications for general medical care (Foy et al., 2014; Seden et al., 2017).

Previous studies conducted in different countries have shown that the prevalence of potential DDIs involving ARV drugs ranges from 18% to 54%, and 0.2% to 5.6% of DDIs involved a contraindicated combination (Seden et al., 2015; Ranzani et al., 2018; Siefried et al., 2018; Schlaeppi et al., 2019). DDIs in HIV populations with multimorbidity factors are inevitable to a large extent and are associated with decreased efficacy, side effects, and suboptimal adherence (Siefried et al., 2018). There are limited data available that address the prevalence of DDIs in resource-limited countries, including mainland China which is likely to face specific risk factors for DDIs. Despite an improvement in HIV screening, among persons newly diagnosed with HIV infection, up to 40% had advanced HIV as defined by World Health Organization (WHO) at the time of their diagnosis in China; the late diagnosis often means experiencing more opportunistic infections (Luo et al., 2016; Pang et al., 2018), having mental health problems (Huang et al., 2018) and consequently implies using more co-medications. In China, as the health systems probably are weak and the patients monitoring sparse, the cases of DDIs may pass unnoticed. With the recent introduction of newer ARVs, Chinese antiretroviral therapy guidelines have changed significantly, which renders the true prevalence of DDIs unknown. Therefore, it is crucial to categorize the frequent interactions of clinical importance.

The objective of this study was to evaluate the prevalence and type of potential ARV DDIs in PLWH through a sizeable multi-center survey in China. Furthermore we identified several factors associated with potential DDIs.

## Patients and Methods

### Study Design and Data Collection

In this cross-sectional multi-center study, we aimed to estimate the proportion of DDIs among PLWH, which was reported as 18% to 54% (Seden et al., 2015; Ranzani et al., 2018; Siefried et al., 2018; Schlaeppi et al., 2019) in previous studies. We estimated the required sample size setting DDIs proportion as 36% (representing the mean between 18% and 54%) and 12% as relative error (i.e. 4.23% as absolute error) using PASS software version 15 (Newcombe, 1998). Consequently, we needed N=475 at least (with 20% inflated sample size estimated at 594 PLWH); therefore, we decided to round to the nearest whole number 600. The HIV treatment centers were chosen based on their reported high prevalence of PLWH (Ding et al., 2019) and/or data availability.

A total of 20 Chinese provinces agreed to join the survey. In each selected province, 1 designated HIV treatment hospital was chosen based on the willingness of their leaders to participate. Concretely, our study covered nearly two-thirds area of China, including the eastern, central, and southern regions. From these 20 hospitals, we randomly selected “30 patients per hospital” from the available database to fulfill the requirement of 600 patients initially calculated. The geographical locations of the study population are shown in **Figure S1** (**Supplementary Material**).

HIV-treatment naïve individuals were defined as ART initiating patients within one month of this survey. At the routine visit of each participant, all currently prescribed drugs were recorded, including prescriptions started before the initiation of ART, which were continued during ART. Polypharmacy was defined as being on ≥5 non-HIV drugs following the method used in recent publications (Courlet et al., 2019; Lopez-Centeno et al., 2019). Sponsored HIV medication was defined as free government-sponsored antiretroviral regimens in China, which currently includes NRTIs (tenofovir, zidovudine, abacavir lamivudine), NNRTIs (efavirenz, nevirapine), and PIs (lopinavir/ritonavir)(Cao et al., 2020). This study was reviewed and approved by the Beijing Youan Hospital institutional review board, which is the leading research institute. Written informed consent was provided by each recruited participant. Their demographics information and prescribed medications were recorded. All the medications were classified and grouped using the anatomical, therapeutic, and chemical (ATC) classification system provided online (www.whocc.no).

For each participant, the most recent prescription was screened to assess the presence or not of DDIs using the University of Liverpool HIV drug interaction database (www.hiv-druginteractions.org, accessed on 20 December 2019), which uses a “traffic light” system. We interpreted the results as following: Green-flags show the absence of anticipated interaction; yellow-flags indicate a potential interaction of weak intensity not requiring additional monitoring/action or dosage adjustment; orange-flags for those who might require dosage adjustment, close monitoring, or timing of administration modification to minimize possible clinical consequences; and red-flags reveal contraindicated combinations, potentially leading to serious adverse events or an impaired efficacy. In this study, we only assessed the interactions between ARVs and non-HIV co-medications. Each drug (including each of those contained in co-formulations) was considered as 1 medication.

### Statistical Analyses

Categorical variables were reported as frequencies (percentages); continuous variables were presented as median (interquartile range) if non-normal distribution or mean (standard deviation) if normal distribution. Univariate and multivariate logistic regression models were used to examine the factors associated with the presence of DDIs. “Antiretroviral Drug Class” was not a categorical variable itself but consist of five independent binary variables, i.e. NRTIs, NNRTIs, boosted PIs, boosted integrase inhibitors (INSTIs) and unboosted INSTIs. There was a mutual adjustment for all ARVs simultaneously in the multivariable model. The five most common opportunistic infections and non-opportunistic comorbidities were selected for inclusion. The inclusion of the top five comorbidities in multivariable model is a post-hoc decision based on survey result. In the HIV literature, the aging population is usually defined as HIV-infected individuals aged ≥ 50 years old (Smith and Flexner, 2017; Ranzani et al., 2018), which means more comorbidities and potentially more DDIs for health providers to manage. Thus, we decided to convert age to a binary categorical variable.

The strength of the associations was measured as odds ratio (OR) and adjusted OR (aOR) with 95% confidence interval (95% CI). A two-tailed *P* value < 0.05 was considered statistically significant. All analyses were performed using SAS version 9.4 software (SAS Institute, Cary, NC, USA).

## Results

### Patient Characteristics and Treatments

A total of 600 PLWH were included; their mean age was 41.2 (± 13.9) years and 81.7% were male. 41.2% (247/600) were treatment-naïve and 63.2% (379/600) were receiving free ARVs (sponsored HIV medication), as recommended by China's National Free Antiretroviral Treatment Program (Ding et al., 2019; Cao et al., 2020). ARV medication profiles revealed that NNRTIs with 2NRTIs regimen was used in 375 participants (62.5%), 125 (20.8%) were on an unboosted INSTIs with 2NRTIs regimen, followed by 59 (9.8%) on a PIs with 2NRTIs regimen. The least used ARVs were boosted INSTIs with 2NRTIs regimen, other non-INSTIs-containing regimens (without 2 NRTIs), and other INSTIs-containing regimens (without 2 NRTIs) found in 20 (3.3%), 12 (2%), and 9 (1.5%) individuals respectively. Dyslipidemia (111, 18.5%), insomnia (88, 14.7%), hypertension (86, 14.3%), gastritis, gastroesophageal reflux disease (36, 6%), and coronary heart disease (33, 5.5%) were the most frequent comorbidities. Fungal infection (127, 21.2%), pneumocystis pneumonia (107, 17.8%), tuberculosis (86, 14.3%), cytomegalovirus infection (46, 7.7%), *Herpes simplex* virus infection (39, 6.5%), and *Mycobacterium avium* complex infection (26, 4.3%) were the most frequent opportunistic infections. Overall, 511 (85.2%) participants were prescribed at least one non-HIV medication along with their ARV regimen, and a low rate of polypharmacy (4.7%) was observed. The details on the demographic and clinical characteristics are shown in **Table 1**.

**Table 1:** Characteristics of the study population.

Variables	Total (n = 600)
Age (year), mean ± SD	41.2 ± 13.9
Age year, (%)	
18–29	137 (22.8)
30–49	283 (47.2)
≥ 50	180 (30.0)
Males, n (%)	490 (81.7)
HIV treatment-naive, n (%)	247 (41.2)
Sponsored HIV medication, n (%)	379 (63.2)
Inpatients, n (%)	100 (16.6)
ARV regimens, n (%)	
2NRTIs + NNRTIs	375 (62.5)
2NRTIs + PIs	59 (9.8)
2NRTIs + unboosted INSTIs	125 (20.8)
2NRTIs + boosted INSTIs	20 (3.3)
other INSTI-containing regimens	9 (1.5)
other non-INSTI-containing regimens	12 (2.0)
Comorbidities, n (%)	
Dyslipidemia	111 (18.5)
Insomnia	88 (14.7)
Hypertension	86 (14.3)
Gastritis, gastroesophageal reflux disease	36 (6.0)
Coronary heart disease	33 (5.5)
Diabetes	22 (3.7)
Arrhythmia	12 (2.0)
Cancer	10 (1.7)
Epilepsy	4 (0.7)
Pulmonary arterial hypertension	2 (0.3)
Opportunistic infections, n (%)	
Fungal infection	127 (21.2)
Pneumocystis pneumonia	107 (17.8)
Tuberculosis	86 (14.3)
Cytomegalovirus infection	46 (7.7)
*Herpes simplex* virus infection	39 (6.5)
*Mycobacterium avium* complex infection	26 (4.3)
Varicella-Zoster virus infection	24 (4.0)
Toxoplasmic encephalitis	12 (2.0)
Number of comedications, n(%)	
0	89 (14.8)
1–4	483 (80.5)
≥ 5	28 (4.7)

SD, standard deviation; ARV, antiretroviral; NRTIs, nucleoside reverse transcriptase inhibitors; NNRTIs, non-nucleoside reverse transcriptase inhibitors; PIs, protease inhibitors; INSTIs, integrase inhibitors.

Comedications was defined as the use of non-HIV medications.

### Prevalence and Type of DDIs by ARV and Non-ARV Drug Classes

**Figure 1:**
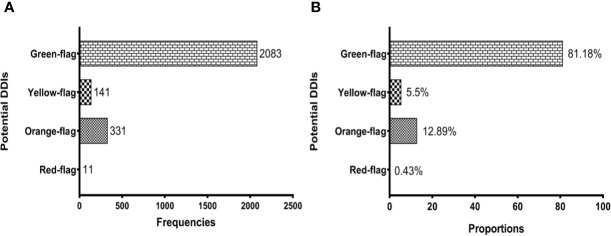
Potential drug-drug interactions (DDIs) among people living with HIV. **(A)** shows the prevalence of DDIs according to frequencies and **(B)** reflects their prevalence based on proportions. Potential DDIs between antiretroviral and non-antiretroviral drugs are represented with different shades according to the severity of potential DDIs: Red-flag (contraindicated), Orange-flag (potential clinical relevance requiring dosage adjustment or close clinical monitoring), Yellow-flag (weak clinical significance), and Green-flag (no interaction).

Overall, 2,566 potential DDIs were identified. Based on the Liverpool HIV drug interaction database predictions, we reported the following prevalence: 0.43% (11/2,566) of red flags, 12.89% (311/2,566) of orange flags, 5.5% (141/2,566) of yellow flags, and 81.18% (2,083/2,566) of green flags (**Figure 1**). A detailed description of the 2,566 potential DDIs according to anchor antiretroviral drugs and non-antiretroviral medications are shown in **Table 2**. The prevalence of red-flag DDIs was 0.19% for NNRTIs, 0.19% for boosted PIs, 0.04% for boosted INSTIs, 0% for NRTIs and unboosted INSTIs. The most frequent red-flag DDIs involved antiinfectives for systemic use (0.43%, 11/2,566), followed by cardiovascular system drugs (0.04%, 1/2,566).

**Table 2:** Prevalence of potential drug-drug interactions according to antiretroviral and non-antiretroviral medications in people living with HIV.

		Red-flag		Orange-flag		Yellow-flag		Green-flag	
		N	%	N	%	N	%	N	%
Antiretroviral drugs									
	NRTIs	0	0.00	87	3.39	74	2.88	1450	56.51
	NNRTIs	5	0.19	177	6.90	56	2.18	363	14.15
	Boosted PIs	5	0.19	48	1.87	7	0.27	39	1.52
	Unboosted INSTIs	0	0.00	12	0.47	0	0.00	220	8.57
	Boosted INSTIs	1	0.04	7	0.27	4	0.16	11	0.43
Non-antiretroviral drugs (ATC code)									
	Alimentary tract and metabolism	0	0.00	9	0.35	0	0.00	143	5.57
	Antiinfectives for systemic use	10	0.39	158	6.16	107	4.17	1085	42.28
	Antibacterials for systemic use	1	0.04	66	2.57	70	2.73	624	24.32
	Antimycobacterials	8	0.31	14	0.55	29	1.13	151	5.88
	Antimycotics for systemic use	1	0.04	62	2.42	0	0.00	227	8.85
	Antivirals for systemic use	0	0.00	16	0.62	8	0.31	83	3.23
	Antineoplastic and immunomodulating agents	0	0.00	0	0.00	0	0.00	3	0.12
	Antineoplastic agents	0	0.00	0	0.00	0	0.00	3	0.12
	Blood and blood forming organs	0	0.00	3	0.12	0	0.00	9	0.35
	Antithrombotic agents	0	0.00	3	0.12	0	0.00	9	0.35
	Cardiovascular system	1	0.04	97	3.78	28	1.09	588	22.92
	Agents acting on the renin-angiotensin system	0	0.00	1	0.04	11	0.43	163	6.35
	Alpha blocking agents	0	0.00	0	0.00	0	0.00	0	0.00
	Beta blocking agents	0	0.00	2	0.08	4	0.16	46	1.79
	Calcium channel blockers	0	0.00	17	0.66	1	0.04	64	2.49
	Diuretics	0	0.00	0	0.00	0	0.00	24	0.94
	Lipid modifying agents	1	0.04	77	3.00	12	0.47	288	11.22
	Vasodilators used in cardiac diseases	0	0.00	0	0.00	0	0.00	3	0.12
	Dermatologicals	0	0.00	0	0.00	0	0.00	3	0.12
	Antifungals for dermatological use	0	0.00	0	0.00	0	0.00	3	0.12
	Genito urinary system and sex hormones	0	0.00	1	0.04	0	0.00	5	0.19
	Drugs used in benign prostatic hypertrophy	0	0.00	0	0.00	0	0.00	3	0.12
	Drugs used in erectile dysfunction	0	0.00	1	0.04	0	0.00	2	0.08
	Nervous system	0	0.00	50	1.95	4	0.16	166	6.47
	Antidepressants	0	0.00	1	0.04	4	0.16	13	0.51
	Antiepileptics	0	0.00	4	0.16	0	0.00	11	0.43
	Antipsychotics	0	0.00	7	0.27	0	0.00	13	0.51
	Anxiolytics	0	0.00	37	1.44	0	0.00	118	4.60
	Hypnotics and sedatives	0	0.00	1	0.04	0	0.00	11	0.43
	Respiratory system	0	0.00	2	0.08	2	0.08	22	0.86
	Antihistamines for systemic use	0	0.00	0	0.00	1	0.04	7	0.27
	Drugs for obstructive airway diseases	0	0.00	2	0.08	1	0.04	15	0.58
	Systemic hormonal	0	0.00	1	0.04	0	0.00	2	0.08
	Thyroid therapy	0	0.00	1	0.04	0	0.00	2	0.08

NRTIs, nucleoside reverse transcriptase inhibitors; NNRTIs, non-nucleoside reverse transcriptase inhibitors; PIs, protease inhibitors; INSTIs, integrase inhibitors; ATC, anatomical therapeutic chemical classification system.

Red-flag: contraindicated; Orange-flag: potential clinical relevance requiring dosage/timing adjustment or close monitoring; Yellow-flag: weak clinical significance; Green-flag: no anticipated interaction.

non-ART drugs not included in anatomical therapeutic chemical classification system (ATC) code (i.e., Chinese herb,…) were included, thus orange and green flag numbers are not equal in ARV vs non-ARV drugs.

The most encountered ARVs in orange-flags were NNRTIs (6.90%), followed by NRTIs (3.39%) and boosted PIs (1.87%). The non-ARV medications most frequently involved in orange-flags were antiinfectives for systemic use (6.08%, 158/2,566), followed by cardiovascular system drugs (3.78%, 97/2,566), nervous system drugs (1.95%, 50/2,566), and alimentary tract and metabolism drugs (0.35%, 9/2,566).

**Figure 2:**
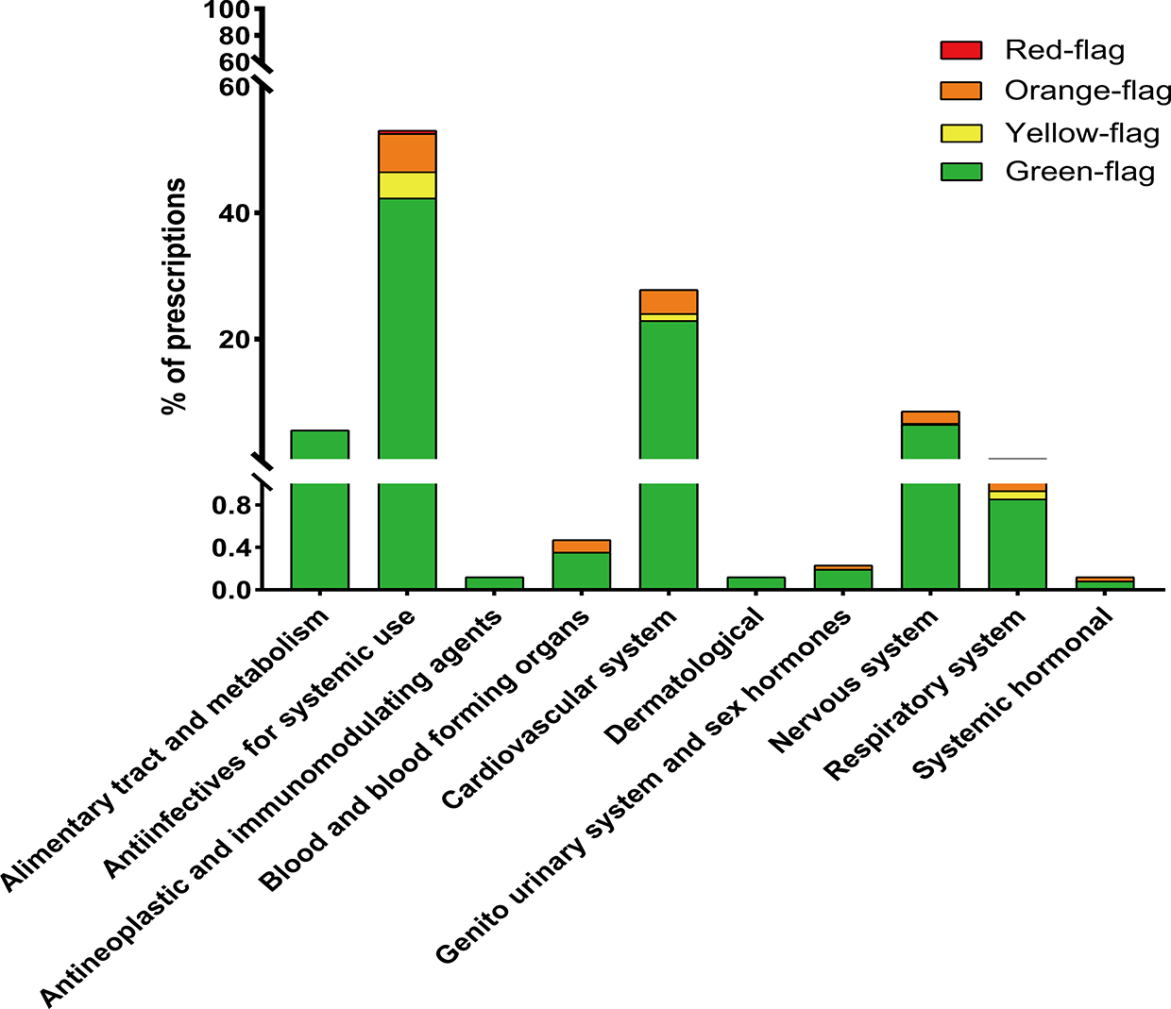
Co-medications sorted according to the anatomical therapeutic chemical classification system and prevalence of DDIs within each therapeutic class.

The non-ARV co-medications taken by our considered participants are outlined in **Figure 2**, and classified according to the ATC system. Among the prescriptions with co-medications, the majority concerned antiinfectives for systemic use (53.04%). Then, cardiovascular system drugs, nervous system drugs, and alimentary tract and metabolism drugs were noted at 27.83%, 8.57%, and 5.92%, respectively. The proportions of PLWH with red-flags, orange-flags, yellow-flags, and green-flags among the participants with non-ARV co-medications are represented with different shades in the corresponding therapeutic class.

### Factors Associated With Drug–Drug Interactions

**Table 3** shows the results of the univariate and multivariate logistic regression analyses to identify the risk factors associated with clinically significant DDIs (red- and orange-flagged co-medications). Factors independently associated with increased risk of DDIs included treatment with boosted PIs (aOR, 27.03; 95% CI, 11.32–64.54; *p* < 0.0001), boosted INSTIs (aOR, 13.69; 95% CI, 4.29–43.69; *p* < 0.0001), and NNRTIs (aOR, 8.09; 95% CI, 3.97–16.51; *p* < 0.0001) as anchor ARVs; as well as treatments associated with the following non-ARV medications: antiinfectives for systemic use (aOR, 3.59; 95% CI, 2.48–5.20; *p* < 0.0001), cardiovascular system drugs (aOR, 3.35; 95% CI, 1.96–5.70; *p* < 0.0001), nervous system drugs (aOR, 3.99; 95% CI, 2.1–7.58; *p* < 0.0001). The other factors that were independently associated with an increased risk were fungal infection (aOR, 1.68; 95% CI, 1.15–2.45; *p* = 0.0071) and *Herpes simplex* virus infection (aOR, 2.11; 95% CI, 1.11–4.02; *p* = 0.0231). The only factor independently associated with reduced risk of DDIs was hypertension (aOR, 0.54; 95% CI, 0.31–0.94; *p* = 0.0297). Factors such as gender, age, inpatient status, sponsored HIV medication, and polypharmacy did not affect the risk of DDIs.

**Table 3:** Factors associated with potential drug-drug interactions.

Variables	Univariate		Multivariable	
	OR (95% CI)	*P* value	aOR (95% CI)	*P* value
Older Adults (**≥** 50 years)	1.33 (1.01–1.75)	0.0393	1.35 (0.95–1.92)	0.0978
Male gender	1.00 (0.72–139)	0.9932	1.12 (0.75–1.67)	0.5706
Inpatients	0.91 (0.64–1.30)	0.6063	1.20 (0.78–1.84)	0.4002
Sponsored HIV medication	1.23 (0.93–1.63)	0.1557	0.95 (0.66–1.38)	0.7925
Polypharmacy	2.44 (1.44–4.12)	0.0009	0.7 (0.33–1.50)	0.3571
Antiretroviral Drug Class				
NRTIs	0.18 (0.14–0.24)	<0.0001	0.99 (0.49–1.98)	0.9699
NNRTIs	4.56 (3.47–6.00)	<0.0001	8.09 (3.97–16.51)	<0.0001
Boosted PIs	6.97 (4.23–11.47)	<0.0001	27.03 (11.32–64.54)	<0.0001
Boosted INSTIs	3.31 (1.34–8.17)	0.0095	13.69 (4.29–43.69)	<0.0001
Unboosted INSTIs	0.44 (0.24–0.81)	0.0078	—	—
Non-antiretroviral drugs (ATC code)				
Alimentary tract and metabolism	1.68 (1.09–2.59)	0.02	1.42 (0.79–2.55)	0.2436
Antiinfectives for systemic use	2.43 (1.83–3.22)	<0.0001	3.59 (2.48–5.20)	<0.0001
Antineoplastic and immunomodulating agents	<0.001 (< 0.001 to >999)	0.9836	<0.001 (< 0.001 to >999)	0.991
Blood and blood forming organs	1.63 (0.44–6.05)	0.4662	0.77 (0.13–4.46)	0.7682
Cardiovascular system	2.04 (1.56–2.67)	<0.0001	3.35 (1.96–5.70)	<0.0001
Dermatologicals	<0.001 (< 0.001 to >999)	0.9836	<0.001 (< 0.001 to >999)	0.9884
Genito urinary system and sex hormones	2.44 (0.22–26.95)	0.4681	3.65 (0.22–59.70)	0.3633
Nervous system	2.32 (1.67–3.22)	<0.0001	3.99 (2.1–7.58)	<0.0001
Respiratory system	0.25 (0.03–1.90)	0.1814	0.59 (0.07–5.14)	0.6346
Systemic hormonal	2.44 (0.22–26.95)	0.4681	13.78 (0.86–220.91)	0.0638
Comorbidities/opportunistic infections				
Dyslipidemia	1.83 (1.36–2.47)	<0.0001	1.11 (0.68–1.81)	0.6844
Insomnia	1.79 (1.29–2.50)	0.0006	1.00 (0.54–1.86)	0.9917
Hypertension	1.34 (0.94–1.91)	0.1014	0.54 (0.31–0.94)	0.0297
Gastritis, gastroesophageal reflux disease	1.25 (0.74–2.10)	0.4042	0.98 (0.51–1.87)	0.9509
Coronary heart disease	1.76 (1.05–2.94)	0.0316	0.68 (0.33–1.36)	0.2723
Fungal infection	1.78 (1.34–2.38)	<0.0001	1.68 (1.15–2.45)	0.0071
Pneumocystis pneumonia	0.66 (0.46–0.96)	0.0304	0.66 (0.41–1.05)	0.0787
Tuberculosis	0.91 (0.62–1.32)	0.6041	0.81 (0.50–1.30)	0.3847
*Herpes simplex* virus infection	1.86 (1.11–3.11)	0.0194	2.11 (1.11–4.02)	0.0231
Cytomegalovirus infection	1.38 (0.88–2.15)	0.162	0.82 (0.46–1.47)	0.504

OR, odds ratio; aOR, adjusted odds ratio; CI, confidence interval; NRTIs, nucleoside reverse transcriptase inhibitors; NNRTIs, non-nucleoside reverse transcriptase inhibitors; PIs, protease inhibitors; INSTIs, integrase inhibitors; ATC, anatomical therapeutic chemical classification system.

## Discussion

To our knowledge, this study is the first in which potential drug-drug interactions involving PLWH in China is addressed. Most of the published studies reporting the prevalence of potential interactions among PLWH were limited to hospital settings (Baecke et al., 2017; Park et al., 2018) or specific groups such as older people (Ranzani et al., 2018; Livio and Marzolini, 2019). In this sample of PLWH, 85.2% took a concomitant medication and 2,566 potential DDIs were identified. 0.43% of DDIs in PLWH receiving ART involved contraindicated combinations. This prevalence was also found in a cohort study performed in Uganda (Seden et al., 2015), but inferior to those reported in other articles (Holtzman et al., 2013; Ranzani et al., 2018; Lopez-Centeno et al., 2019) partly because of the methods of evaluation of DDIs and the use of co-medications.

The mean age of the study population was 41.2 years, and 30% of them were ≥ 50 years (designated as “aging population” in HIV-related literature). More than half of the study population (67%) had at least one known comorbidity, which is as high as other cohorts that have found at least one comorbidity in 56% (Siefried et al., 2018) or 70% (Hasse et al., 2011) of PLWH over 50 years old. The age-related comorbidities burden leads inevitably to polypharmacy (Ranzani et al., 2018; Lopez-Centeno et al., 2019), which increases the chance of DDIs. However, in contrary to previous studies, older adults (≥ 50 years) and polypharmacy were not risk factors associated with potential DDIs in our study. Probably, the age distributing status or the lack of power subsequent to the small number of participants could explain our observations.

According to the Liverpool classification system, yellow-flag is probably not very intense and unlikely to require any specific intervention. As a result, we defined orange- and red-flags as clinically significant DDIs. In multivariable analysis, as expected and in line with previous studies (Marzolini et al., 2010; Siefried et al., 2018; Lopes et al., 2020), the factors that independently increased the risk of DDIs were boosted PIs, boosted INSTIs, and NNRTIs-containing ARV regimens. During the study period, available free ARVs for first-line and second-line regimens (i.e., efavirenz, lopinavir/ritonavir) in China were among the drugs that are the most susceptible to DDIs (Aids et al., 2018; Ding et al., 2019). In China, although the introduction of more recent agents is delayed due to their availability, they are currently recommended first-line drugs (Aids et al., 2018) such as dolutegravir, dolutegravir-abacavir-lamivudine or elvitegravir-cobicistat-tenofovir alafenamide-emtricitabine. Many of them often consist of fixed-dose combinations, so the management of DDIs could be more complicated. In addition, increased risk of significant DDIs was noted in participants having fungal or *Herpes simplex* virus infection. Surprisingly, hypertension was associated with a reduced risk of DDIs. Further studies are needed to assess the potency of our analysis.

Herein, clinically significant DDIs mostly concerned antiinfectives for systemic use. Notably, rifampicin was involved as contraindicated, which is in accordance with previous studies conducted in resource-limited settings (Kigen et al., 2011; Gimeno-Gracia et al., 2015; Schlaeppi et al., 2019). The second most prevalent DDIs were noted with cardiovascular system drugs, which is explained by the aging of the population of PLWH. Cardiovascular drugs and nervous system drugs were the most represented therapeutic classes in older and younger PLWH, respectively (Marzolini et al., 2010; Kamal et al., 2018; Courlet et al., 2019). In line with these reports, our results are showing that the third most prevalent DDIs involved nervous system drugs. It is worth noting that psychiatric illness, including anxiety and depression, is often prevalent in the HIV-infected population across China (Huang et al., 2018). Attention to DDIs regarding these drugs may help improving patients safety considerably.

Despites the observations reported, our study has several limitations. On one hand, no control group of uninfected individuals was included. Then, the clinical relevance and patient outcomes were unknown. Thus, whether appropriate management such as close monitoring or dose modifications were made should be longitudinally evaluated to determine the clinical importance of the DDIs. On the other hand, the use of over-the-counter (OTC) drugs may have been not recorded in clinical notes. OTC drugs are often supplied to patients outside the clinic and may, therefore, escape the attention or approval of clinicians. They may significantly interact with ARVs and, consequently, the real burden of DDIs could be underestimated. Supplements such as multivitamins containing magnesium, iron, or calcium may interact with INSTIs, which have been reported to account for 3.7% of all the DDIs in a recent report (Siefried et al., 2018). In addition, the adherence to treatment monitoring was not performed. Finally, the current results should be further verified in larger-scale studies.

Awareness of prescribers on DDIs and knowledge of highly prevalent DDIs are essential for safe prescribing and drug use. In China, physician recognition of DDIs involving ARVs may be incomplete or low, as reported in a UK study (Evans-Jones et al., 2010). Although there are several excellent resources available to guide providers in the management of drug interactions involving antiretroviral medications, the clinicians are probably less familiar with these tools. Focused and intense education should be carried out to increase DDIs awareness in order to achieve effective treatment of HIV in China. At the same time, DDIs awareness can facilitate the introduction of interventions and guidance on interactions management. Medical providers should always conduct a thorough medication history at each visit regarding the use of all medications including: prescription, over-the-counter, herbal, and recreational drugs. Furthermore, they should consider potential interactions before prescribing a new medication.

To conclude, DDIs in PLWH receiving ART together with treatment for comorbidities can be expected in a considerable proportion across China. Understanding the DDIs profile in the population can help medical providers to develop practical approaches for their recognition and management.

## Data Availability Statement

The raw data required to support the findings of this study cannot be shared at this time as the data also forms part of an ongoing study.

## Ethics Statement

The study was conducted in accordance with the Declaration of Helsinki. Written Informed consent was obtained from all patients. This study was reviewed and approved by the Beijing Youan Hospital institutional review board, which is the leading research institute for this study.

## Author Contributions

HX and LG contributed equally as co-first authors. XH, TZ, and HW contributed to the design of this study. HX, TZ, HW, and PM contributed to data collection. HX, LG, XG, SZ, and XH analyzed the data and wrote the article. All authors read and approved the final manuscript.

## Funding

This study was funded by the 13th Five-year National Major Project for HIV/AIDS and Hepatitis B Control and Prevention, Chinese Ministry of Science and Technology (2017ZX10202102005004), the National science and technology major project of China during the 13th Five-year plan period (2017ZX10201101), the National Natural Science Foundation of China (No. 81701984), and the Beijing Excellent Talent Plan (2018000021223ZK04).

## Conflict of Interest

The authors declare that the research was conducted in the absence of any commercial or financial relationships that could be construed as a potential conflict of interest.

## Supplementary Material

Supplementary Figure S1 Geographical distribution of the participating centers.
