# Supplementary figures and images for: The Challenge of Potential Drug–Drug Interactions Among People Living With HIV on Antiretroviral Therapy: A Cross-Sectional Study in Selected Provinces in China
Source: Front Pharmacol. 2020 May 27;11:800. doi: 10.3389/fphar.2020.00800 (PMC7266979; doi:10.3389/fphar.2020.00800)

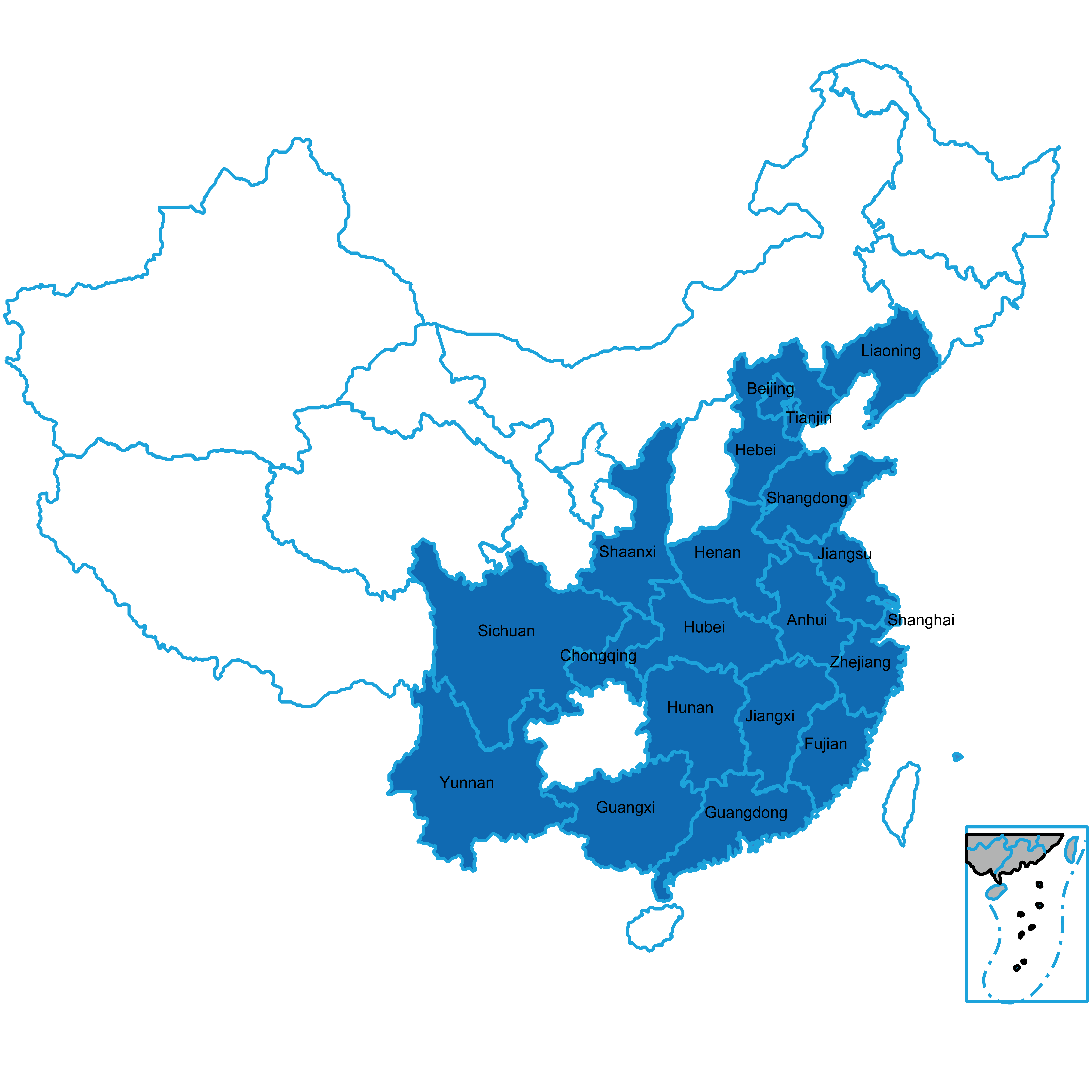

Supplement: Supplementary Figure S1 — Geographical distribution of the participating centers. [file Image_1.tif]
